# Evolutionary origin of highly repetitive plastid genomes within the clover genus (*Trifolium*)

**DOI:** 10.1186/s12862-014-0228-6

**Published:** 2014-11-18

**Authors:** Saemundur Sveinsson, Quentin Cronk

**Affiliations:** Department of Botany and Biodiversity Research Centre, University of British Columbia, 6270 University Boulevard, Vancouver, BC V6T 1Z4 Canada

**Keywords:** Trifolium, Clover, Plastome evolution, Plastid genome evolution, Repetitive DNA, Fabaceae

## Abstract

**Background:**

Some clover species, particularly *Trifolium subterraneum*, have previously been reported to have highly unusual plastomes, relative to closely related legumes, enlarged with many duplications, gene losses and the presence of DNA unique to *Trifolium*, which may represent horizontal transfer. In order to pinpoint the evolutionary origin of this phenomenon within the genus *Trifolium*, we sequenced and assembled the plastomes of eight additional *Trifolium* species widely sampled from across the genus.

**Results:**

The *Trifolium* plastomes fell into two groups: those of *Trifolium boissieri*, *T. strictum* and *T. glanduliferum* (representing subgenus *Chronosemium* and subg. *Trifolium* section Paramesus) were tractable, assembled readily and were not unusual in the general context of Fabeae plastomes. The other *Trifolium* species (“core *Trifolium*”) proved refractory to assembly mainly because of numerous short duplications. These species form a single clade, which we call the “refractory clade” (comprising subg, *Trifolium* sections Lupinaster, Trifolium, Trichocephalum, Vesicastrum and Trifoliastrum). The characteristics of the refractory clade are the presence of numerous short duplications and 7-15% longer genomes than the tractable species. Molecular dating estimates that the origin of the most recent common ancestor (MRCA) of the refractory clade is approximately 13.1 million years ago (MYA). This is considerably younger than the estimated MRCA ages of *Trifolium* (c. 18.6 MYA) and *Trifolium* subg. Trifolium (16.1 MYA).

**Conclusions:**

We conclude that the unusual repetitive plastome type previously characterized in *Trifolium subterraneum* had a single origin within *Trifolium* and is characteristic of most (but not all) species of subgenus *Trifolium*. It appears that an ancestral plastome within *Trifolium* underwent an evolutionary change resulting in plastomes that either actively promoted, were permissive to, or were unable to control, duplications within the genome. The precise mechanism of this important change in the mode and tempo of plastome evolution deserves further investigation.

**Electronic supplementary material:**

The online version of this article (doi:10.1186/s12862-014-0228-6) contains supplementary material, which is available to authorized users.

## Background

The increased availability and lowered costs of various massive parallel sequencing technologies have resulted in a dramatic expansion of fully sequenced plastid genomes [1]. There are currently 512 plastome sequences listed at NCBI’s Organelle Genome Resource website (http://tinyurl.com/ncbi-plastid-genomes, 17 April 2014) and half of them have been made available since 2012. Their structure, gene order and gene content is generally highly conserved across most flowering plants, where most plastomes have two copies of a highly conserved inverted repeat (IR) and two conserved single copy regions (see [2]). However, there are some well known and striking exceptions from these conserved plastome structures in several photosynthetic angiosperm lineages, such as the Geraniaceae [3] and Campanulaceae [4].

Considerable advances have recently been made in the development of genomic resources for the clover genus (*Trifolium*), with the publication of the genome assembly [5] and analyses of the transcriptome [6] of red clover (*T. pratense*). Subterranean clover (*Trifolium subterraneum*) is known to have an unusual plastid genome structure [7]. Firstly it lacks one copy of the inverted repeat, but that is a character shared with a large group of papilionoid legumes [8], designated the inverted repeat lacking clade (IRLC). Secondly its plastome has undergone more than a dozen rearrangements, i.e. translocations and/or inversions, compared to the plastid genome of *Medicago truncatula* [7]. However, highly rearranged plastomes also occur in the related tribe Fabeae, represented by *Lathyrus sativus* and *Pisum sativum* [9]. Finally, and very unusually, its plastome contains about fourfold the amount of repeated DNA compared to related legume species and is consequently about 20 kb longer [7]. A recent study has shown that this unusual repeat-rich plastome structure is not unique to *T. subterraneum* [10]. However, the extent to which this is a widespread feature of *Trifolium* species, or characteristic of a restricted part of the genus *Trifolium* still needs further investigation. To answer this question, we performed a low coverage whole genome shotgun sequencing of nine strategically sampled *Trifolium* species and were able to assemble eight plastid genomes. These plastomes were then analysed to elucidate the phylogenetic distribution of plastome variation in the genus.

## Results

### Plastome assembly and structural variability

**Figure 1:**
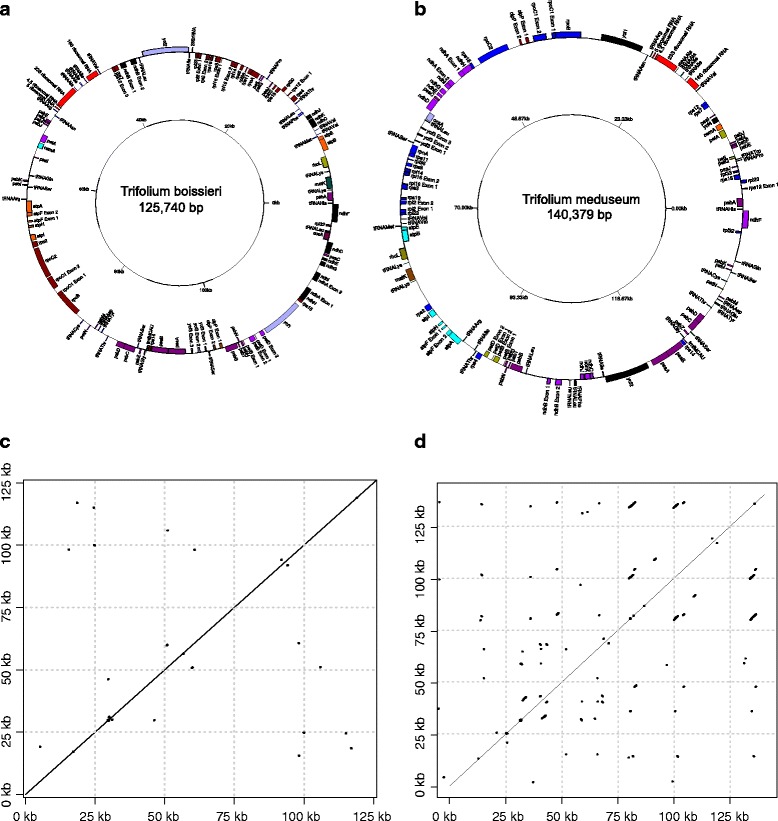
**Gene map of two** ***Trifolium*** **plastid genomes and dotplots illustrating repeated regions.** Gene maps **(a and** **b)** and dotplots **(c and** **d)** of *Trifolium boisseri* **(a and** **c)** and *Trifolium meduseum* **(b and** **d)**. The gene maps shows the postions and translation direction of protein coding genes, tRNAs and rRNAs in the plastid genome. The dotplots illustrate repeated sequences within the plastomes, identified by BLASTN, where aligned sequences are shown as lines. Since the plastomes are aligned to themselves, the entire plastome is represented as a long and uninterrupted diagonal line. Shorter lines and dots, that fall either above or below the long line are repeats.

The plastomes of three sequenced *Trifolium* species, *Trifolium boissieri*, *T. strictum* and *T. glanduliferum*, were tractable, assembled easily and had no unusual structure, at least in the context of related species in the inverted repeat loss clade (IRLC), such as species of the tribe Fabeae (see [9,10]) (Figure 1A and C) [GenBank: KJ788284, KJ788292 and KJ788285]. The remaining six *Trifolium* species had plastome structure similar to *T. subterraneum* (described in [7]), containing several short repetitive regions, which made them refractory to assembly (see Table 1). However, with one exception we were able to assemble all of them successfully, using careful analysis of the paired-end sequences (see below) (Figure 1B and D) [GenBank: KJ788286 - KJ788289 and KJ788291]. The exception is *T. pratense*. The plastome structure of *T. pratense* appears to be highly complex and we were unable to complete a full assembly of it using the methods employed in this study. It is clear that this plastome contains several repeated regions, similar to *T. subterraneum*. We were, however, able to put together three large plastid contigs for *T. pratense*, with a combined length of 121 kb, which were annotated with DOGMA [11] and used for phylogenetic analysis [GenBank: KJ788290]. We generated well supported plastome assemblies for the remaining five “difficult” species and verified them using mapping information from the paired end Illumina reads (see Table 1). We ensured that the entire assembly had sufficient coverage (at least 100x) and that the placement of adjacent plastid regions were supported by the mapping of paired end reads. However it is important to note, that despite these careful quality checks, assembly of some regions that have a particularly high frequency of repeats should remain provisional. Consequently these are deposited on GenBank as “unverified”.

**Table 1:** **Summary Illumina sequencing accessions and plastome assembly information of** ***Trifolium*** **species**

**Species (USDA seed accession) [Section]**	NO trimmed reads (mean length) [SD*]	**Plastome length (repetitive %)**	**GenBank accession**	**Herbarium voucher**
*Medicago truncatula*	NA	124,033 nt	NC_003119	NA
Gaertn. (NA) [NA]		(1.46%)		
*T. aureum* Pollich (NA)	NA	126,970	NC_024035	NA
[Subg. *Chronosemium*]		(5.6%^**^)		
*T. grandiflorum* Schreb. (NA)	NA	125,628	NC_024034	NA
[Subg. *Chronosemium*]		(4.3%^**^)		
*T. subterraneum* L.	NA	144,763 nt	NC_011828	NA
(NA) [Trichocephalum]		(20.71%)		
*T. repens* L. (NA)	NA	132,120	NC_024036	NA
[Trifoliastrum]		(20.7%^**^)		
*Trifolium boisseri* Guss. (PI 369022)	20.5 × 10^6^	125,741 nt	KJ788284*	Sveinsson 14–01 (UBC)
[Subg. *Chronosemium*]	(95.02) [14.08]	(1.05%)		
*T. strictum* L.	16.7 × 10^6^	125,835 nt	KJ788292*	Sveinsson 14–02 (UBC)
(PI 369147) [Paramesus]	(95.13) [13.98]	(0.71%)		
*T. glanduliferum* Boiss. (PI 296666)	11.5 × 10^6^	126,182 nt	KJ788285*	Sveinsson 14–03 (UBC)
[Paramesus]	(95.16) [13.96]	(0.78%)		
*T. lupinaster* L.	24.8 × 10^6^	135,077 nt	KJ788287*	Sveinsson 14–04 (UBC)
(PI 631632) [Lupinaster]	(94.77) [14.33]	(5.98%)		
*T. meduseum* Blanche	18.9 × 10^6^	140,380 nt	KJ788288*	Sveinsson 14–05 (UBC)
ex Boiss. (PI 369049) [Trichocephalum]	(95.14) [14.01]	(12.83%)		
*T. pratense* L. cv.	22.2 × 10^6^	NA	KJ788290*	Sveinsson 14–07 (UBC)
Arlington (G 27569) [Trifolium]	(94.85) [14.29]	(NA)		
*T. hybridum* L.	21.4 × 10^6^	134,881 nt	KJ788286*	Sveinsson 14–08 (UBC)
(PI 634109, as *T. pallescens*) [Vesicastrum]	(95.11) [14.04]	(7.86%)		
*T. semipilosum*	29.1 × 10^6^	138,242 nt	KJ788291*	Sveinsson 14-09(UBC)
Fresen. (PI 262238) [Vesicastrum]	(94.80) [14.33]	(10.55%)		
*T. occidentale* Coombe	18.6 × 10^6^	133,806 nt	KJ788289*	Sveinsson 14–10 (UBC)
(PI 641363) [Trifoliastrum]	(95.35) [13.80]	(4.64%)		

An asterisk marks plastome sequences newly reported in this paper. Only a partial assembly of *T. pratense* was possible with our data (see text for explanation).

*SD: Standard Deviation.

**Estimations from [10].

### Phylogenetic distribution of the refractory species

**Figure 2:**
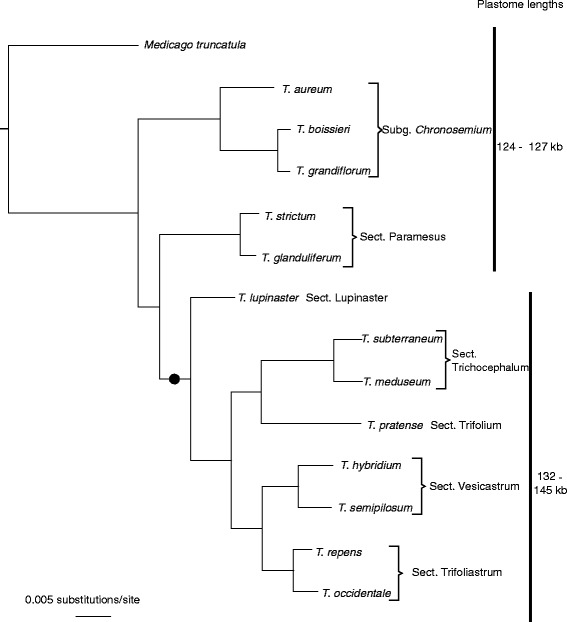
**A maximum likelihood phylogeny of** ***Trifolium*** **.** Phylogenetic relationships among the *Trifolium* species, generated from a concatenated matrix of 58 protein coding plastid genes with a combined aligned length of 48,058 characters. The black dot marks the clade with plastomes refractory to assembly and the black vertical lines indicate the plastome size categories.The age of the most recent common ancestor (MRCA) of the refractory clade is estimated to be 12.4 – 13.8 million years old. All nodes have a 100% bootstrap support.

The six *Trifolium* species with plastomes that were refractory to assembly differed markedly from the three species that were tractable for assembly. Furthermore, the six species and the previously sequenced *T. subterraneum* and *T. repens* all belong to the same clade within *Trifolium*, which we call the “refractory clade”*,* comprising subg. *Trifolium* sections Lupinaster, Trifolium, Tricocephalum, Vesicastrum and Trifoliastrum (see Figure 2).The remaining three species, which have plastomes that assembled readily, are all outside this clade and represent subgenus *Chronosemium* and subgenus *Trifolium* section Paramesus (see Figure 2). Molecular dating analysis of the refractory clade revealed that it originated, i.e. had a most recent common ancestor (MRCA), about 12.4 – 13.8 MYA. The refractory clade has therefore had a considerable amount of time in which to accumulate a high diversity of repeat patterns. This compares to estimated MRCA ages of 16.1 MYA for *Trifolium* subgenus Trifolium and 18.4 MYA for the genus *Trifolium* as a whole. The phylogeny in Figure 2 was inferred using maximum likelihood from a concatenated matrix of 68 protein coding plastid genes, with a combined aligned length of just over 48 kb. All nodes have 100% bootstrap support and the topology is consistent with the most recent phylogenetic treatment of *Trifolium* [12].

### The nature of the duplicated regions

The amount of repetitive sequence in the *Trifolium* plastomes ranged from 0.71% to 20.71 % (see Table 1). This was based on a reciprocal BLASTN search, where each plastome was used as the query and subject (see Methods). Not surprisingly, plastomes belonging to the refractory clade contained about 5–20 times more repetitive DNA than *Trifolium* species in subgenus *Chronosemium* and subgenus *Trifolium* section Paramesus. Repetitive regions larger than 300 bp were subjected to a BLASTX search, in order to determine whether they contained any protein coding regions. A protein database was compiled of all annotated genes from the *Trifolium* plastomes. About 60% of the longer repetitive regions did not show significant (E-value ≤1E-06) similarity to any protein coding regions. The remaining 40% of showed similarity to one of the following plastid genes: *clpP*, *psaJ*, *psbK*, *psbN*, *rpl2*, *rpl23*, *rpoB*, *rps18*, *rps3*, *ycf1*and *ycf3*, indicating that at least partial duplication of genic regions has occurred. Patterns of genic duplication are highly variable, and no genes were found to be duplicated in more than two species. All repetitive gene regions consisted of only a partial reading frame and no duplicated genes were found with the entire reading frame intact in more than one copy. However a small number of gene duplicates appeared to have relatively large portions of open reading frames (ORF) that appeared to be intact (i.e. identical at the protein level to the full length functional copy). A BLASTX search of repetitive regions revealed that *psaJ*, *psbK*, *psbN*, *rpl2*, *ycf1* and *ycf3* had instances where they are 100% intact over 5 – 30% of the length of the corresponding complete ORF.

### Instances of gene loss

The *Trifolium* plastomes have undergone some gene loss. *AccD* appears to missing from plastomes in the following sections of the subgenus *Trifolium*: Trifolium, Tricocephalum, Vesicastrum and Trifoliastrum. This is in agreement with previous reports [7,10]. Our broad phylogenetic sampling shows that the plastid copy of *accD* is also still intact in *Trifolium lupinaster* (section Lupinaster of the subgenus *Trifolium*).

## Discussion

### The Phylogenetic distribution of plastome types

Two types of plastomes were observed among the *Trifolium* species in this study. Three species had no unusual structure in the context of IRLC plastomes, which are known to have numerous rearrangements (see [9,10]). The six remaining species had enlarged, repeat-containing plastome structure similar to *T. subterraneum* and *T. repens* (described in [7,10]). These had several repeated regions and were 7 – 21 kb larger compared to *Medicago truncatula, T. boissieri*, *T. strictum* and *T. glanduliferum* (see Figure 2). Furthermore, we observed a strong phylogenetic clustering of these larger, more repeated plastomes. They comprise a single clade (Figure 2) and, strikingly, all the sampled species of this clade have the unusual repeat-rich plastomes. The North-American section Involucrarium also belongs to this clade (it is sister to section Trifoliastrum [12]), and is therefore likely to have similar “refractory” plastomes, although this has yet to be confirmed. This clade contains about 200 species or roughly 70% of the genus, which has a total of roughly 300 species. The evolutionary success of the clade with the repetitive plastomes leaves no doubt about the functional effectiveness of these plastomes despite their unusual structure.

### Potential causes of genome instability and functional significance of the repeat regions

Some regions within the *T. subterraneum* plastome have been suspected to be of bacterial origin by lateral transfer [7], however those regions are not repetitive. The repetitive regions seem to be of plastid origin, as our BLASTN and BLASTX searches to NCBI’s nucleotide collection (nt/nr) and non-redundant protein sequences (nr), did not indicate any obvious non-plastid sequence. A more recent study found no evidence of any bacterial DNA in plastid genomes of several *Trifolium* species [10]. Repeated sequences have been associated with the plastid rearrangements in other angiosperm lineages, such as the Campanulaceae [4,13] and Geraniaceae [3,14]. However, it is clear that plastomes can be highly rearranged without being highly repetitive, such as *Pisum sativum* and *Lathyrus sativus* (Fabaceae) [9].

It is not yet known what has caused repeated sequences to evolve within the plastomes of the *Trifolium* refractory clade although it is likely that this is under the control of nuclear genes. Plastid reorganization is a common process and there is evidence that reorganized molecules may comprise around 1% of the plastid complement of plant cells [15]. This variation may be caused by either homologous recombination (HR) acting on perfect repeats of >50 bp, or microhomology-mediated break-induced replication (MMBIR) acting on microhomologous repeats of <30 bp [16,17]. Various nuclear genes are known to be important in maintaining plastome stability through recombination control and surveillance, and these are therefore candidate genes for genome instability in certain lineages. The *recA* gene has an important role in homologous recombination and there are plant copies that localize to the chloroplast [18,19]. Plastid-targeted *WHIRLY* genes are known to promote plastome stability, apparently by preventing build up of abnormal molecules produced by MMBIR. *Arabidopsis* plants lacking functional copies of relevant *AtWHY* genes show increased accumulation of abnormal plastid DNA with irregular duplications, deletions and circularization events [16].

Most of these abnormal plastid forms are deleterious and transient, not contributing to plastid evolution in the majority of plant lineages. An important consideration therefore is not the genomic changes that cause an increased frequency of plastome reorganization, but what genome changes allow such major plastome changes to persist without being eliminated as deleterious. It may be that certain plastid genes are particularly sensitive to genome rearrangements and their removal to the nuclear genome makes the plastid genome more permissive to rearrangement. The plastid gene *accD* for instance, known to be essential for plant development [20] and with recombinationally active repeats [21], has been moved to the nucleus independently in two lineages with highly rearranged plastomes: Campanulaceae [22] and *Trifolium* [9,10].

One possible consequence of having repeated sequences in a circular molecule, such as the plastid genome, is that if recombinationally active they could potentially allow intramolecular recombination of the plastome into subgenomic molecules much in the manner of the plant mitochondrion, which is rich in recombinational repeats. This has been suggested as a reason for the repeat richness of certain algal plastomes [23]. The *Trifolium* plastomes are much less rich in repeats than *Chlamydomonas*, or than the mitochondrial genome of land plants, but it is nevertheless interesting to consider the idea that the repeat accumulation in *Trifolium* plastomes is being driven by the advantage of maintaining substochiometric populations of specific ORFs in cellular subpopulations [24].

## Conclusions

Although we have sampled only a relatively small number of species in a large genus, the results are nevertheless striking. We show that the *T. subterraneum*-type plastome, i.e. containing large amounts of repetitive DNA, is phylogenetically restricted to a single core clade of the genus (the “refractory clade”), comprising five of the eight recognized sections within *Trifolium*. Furthermore it is ubiquitously present in all members of this clade in our sample. It is thus reasonable to suppose that this may be the characteristic plastome of this core clade. *Trifolium* is a large genus of c. 300 species, and these five sections contain slightly over 200 species, or about 70% of the genus. There are therefore likely to be abundant exemplars of this unusual plastome type, at varying degrees of evolutionary relatedness, available for future functional and evolutionary studies.

## Methods

### Plant material and Illumina sequencing

Sampling was strategically placed across the genus using the sectional classification of [12]. Total DNA was extracted from fresh leaf material of plants that had been grown from seeds in a greenhouse (at UBC), following a modified version of the CTAB protocol [25]. The seeds were obtained from the United States Department of Agriculture (USDA) National Plant Germplasm System, more specifically the National Temperate Forage Legume Genetic Resources Unit in Prosser, WA. Plants were grown until they flowered, the material was critically determined and herbarium specimens collected (of all but one accession, see Table 1). RNase treatments were performed (cat. 19101, QIAGEN, Germantown, MD) and DNA quality was assessed by visual inspections on 1% agarose gels. Illumina sequencing libraries were constructed from high quality DNA, using the NEXTflexTM DNA sequencing kit (100 bp Paired-End reads) (cat: 5140–02, Bioo Scientific Corp, TX). We followed the manufacturer’s protocol and c. 400 bp DNA fragments were size selected using Agencourt AMPure XpTM magnetic beads (cat. A63880, Beckman Coulter Genomics, MA). Completed libraries were pooled and sequenced on a lane of the Illumina HiSeq-2000 platform.

### Plastid genome assemblies and annotation

Trimmomatic v.0.3 [26] was used to trim and remove low quality Illumina reads, with the following flags: LEADING:20 TRAILING:20 SLIDINGWINDOW:4:15MINLEN:36. High quality reads were used in all subsequent analysis and singlet reads, i.e. reads without a paired end, were discarded. We used the *de novo* method implemented in CLC Genomic Workbench v.7.0.2 to generate assemblies for each species, using the default settings. Contigs of plastid origin were identified by a blast search [27] to the available plastid genome of *Trifolium subterraneum,* published in [7] [GenBank: NC_011828]. These were generally the largest and most highly covered contigs in the *de novo* assembly and always had an E-value of 0 when blasted to the *T. subterraneum* plastome. Regions with nucleotides represented as Ns were manually resolved by retrieving sequence information directly from the quality trimmed reads. For three out of eight species, the *de novo* assembly returned a single large plastid contig. This was not the case for the plastid genome assembly of the remaining six *Trifolium* species, where the *de novo* assembly resulted in about five to 10 plastid contigs. In those cases we decided to extend both ends of the plastid contigs by manually appending sequence from the Illumina reads. The sequence of each contig was extended until an overlap of at least 50 bp could be identified at the end of another contig in the assembly, whereupon the contigs where joined. We identified a few short (1–2 kb) regions which were duplicated and hence could be joined to more than two other contigs in the *de novo* assembly. We resolved positions of these regions by adding them in the plastome assembly as many times as was necessary for all the plastid contigs to be joined in a circle. This methodology worked well for joining all the plastid contigs into a well supported hypothesis for a plastome assembly of species in the refractory clade, deposited on GenBank as unverified whole plastome assemblies. The exception was red clover (*Trifolium pratense*) for which only a partial assembly could be reliably recovered. The quality of each plastome assembly was verified by visually by inspecting a BWA mem pileup, v. 0.7.5a [28], of paired end reads using Tablet v.1.13.12.17 [29]. We ensured that the connections between manually joined contigs were supported by paired-end read mapping. Finally all plastome assemblies were annotated using DOGMA [11]. Maps of plastid genomes were generated using GenomeVX [30] and visually adjusted using Inkscape (www.inkscape.org).

### Identification and analysis of repeated DNA in the plastid genomes

Repeated segments where determined in each of the plastid genomes by a reciprocal BLASTN search [27], using the NCBI’s online BLAST service (http://blast.ncbi.nlm.nih.gov/), where each sequence was used as the query and subject. We used these BLAST outputs to generate dotplots, using R [31]. We decided to analyze repetitive regions that the BLASTN search identified as 300 bp or longer and had an E-value of 0, by subjecting them to a BLASTX search using a custom database of plastid genes from closely related species. A BLASTX similarity cutoff was set by removing all hits with an E-value larger than 1E-06. We furthermore subjected all sequences that did not show significant BLASTX similarity to the *Trifolium* plastid genes, to a BLASTX and BLASTN search to NCBI’s nucleotide collection (nt/nr) and non-redundant protein sequences (nr). In order to estimate the percentage of repetitive sequences within the plastomes, we performed a reciprocal BLASTN search of each plastid genome. Only BLAST hits with an alignment length over 79-bp and E-value of 1E-06 were used in the calculations. To gain an overall view of variation in the plastid genomes used in this study, the genomes were aligned using program MAUVE [32]. This alignment is given in Additional file 1.

### Phylogenetic analysis

Due to the extensive rearrangements observed in the plastomes, we restricted our plastome phylogenetic analysis to protein coding genes. We used a custom phylogenetic pipeline, plast2phy, that extracted protein coding regions from DOGMA annotated plastomes, aligned individual gene with Mafft v. 7.0.5 (−auto flag) [33], trimmed alignment gaps using trimAl v.1.2 (−automated1 flag) [34] and finally generated a concatenated alignment of all genes. The pipeline, Plast2phy, written in Python, is available at https://github.com/saemi/plast2phy. We only included genes that were present in all species and showed no evidence of duplication. Models of base substitution were tested for the concatenated matrix using jModelTest v.2.1.1 [35,36]. Using the Akaike information criterion, we determined the GTR + G + I model optimal for the concatenated plastome alignment. We analyzed the dataset under maximum likelihood (ML) [37], using GARLI [38]. We ran GARLI v. 2.0 with default settings, using ten independent searches and 100 bootstrap replicates. Bootstrap consensus was calculated using SumTrees v. 3.3.1 in the DendroPy package [39]. To obtain an approximate age for nodes on this tree by molecular dating we set the *Medicago*-*Trifolium* divergence at 24.1 MYA [40] and estimated the other node ages under penalized likelihood [41] implemented in r8s v.1.8 [42]. We chose the penalized likelihood algorithm since a likelihood ratio test, using constrained and unconstrained likelihood scores obtained from PAUP* [43], rejected the molecular clock (p <0.001). Confidence intervals were estimated by running dating analysis on 100 bootstrap resampled datasets generated by GARLI and are represented as two times the standard deviation. Trees from phylogenetic analysis were drawn using FigTree v.1.4.0 (http://tree.bio.ed.ac.uk/software/figtree/), rooted with *Medicago truncatula* [GenBank:NC_003119] and visually adjusted using Inkscape (http://www.inkscape.org/).

### Availability of supporting data

The sequence data generated in this study are available in GenBank under the accession numbers KJ788284-KJ788292 (see Table 1). The sequence alignment matrix and the corresponding phylogenetic tree are available in the Dryad Digital Repository (doi:10.5061/dryad.km38g) [44].

## Additional file

Additional file 1:
**Genome alignments of plastomes reported in this paper using MAUVE.**
