# Supplementary figures and images for: Evolutionary origin of highly repetitive plastid genomes within the clover genus (Trifolium)
Source: BMC Evol Biol. 2014 Nov 18;14:228. doi: 10.1186/s12862-014-0228-6 (PMC4241210; doi:10.1186/s12862-014-0228-6)

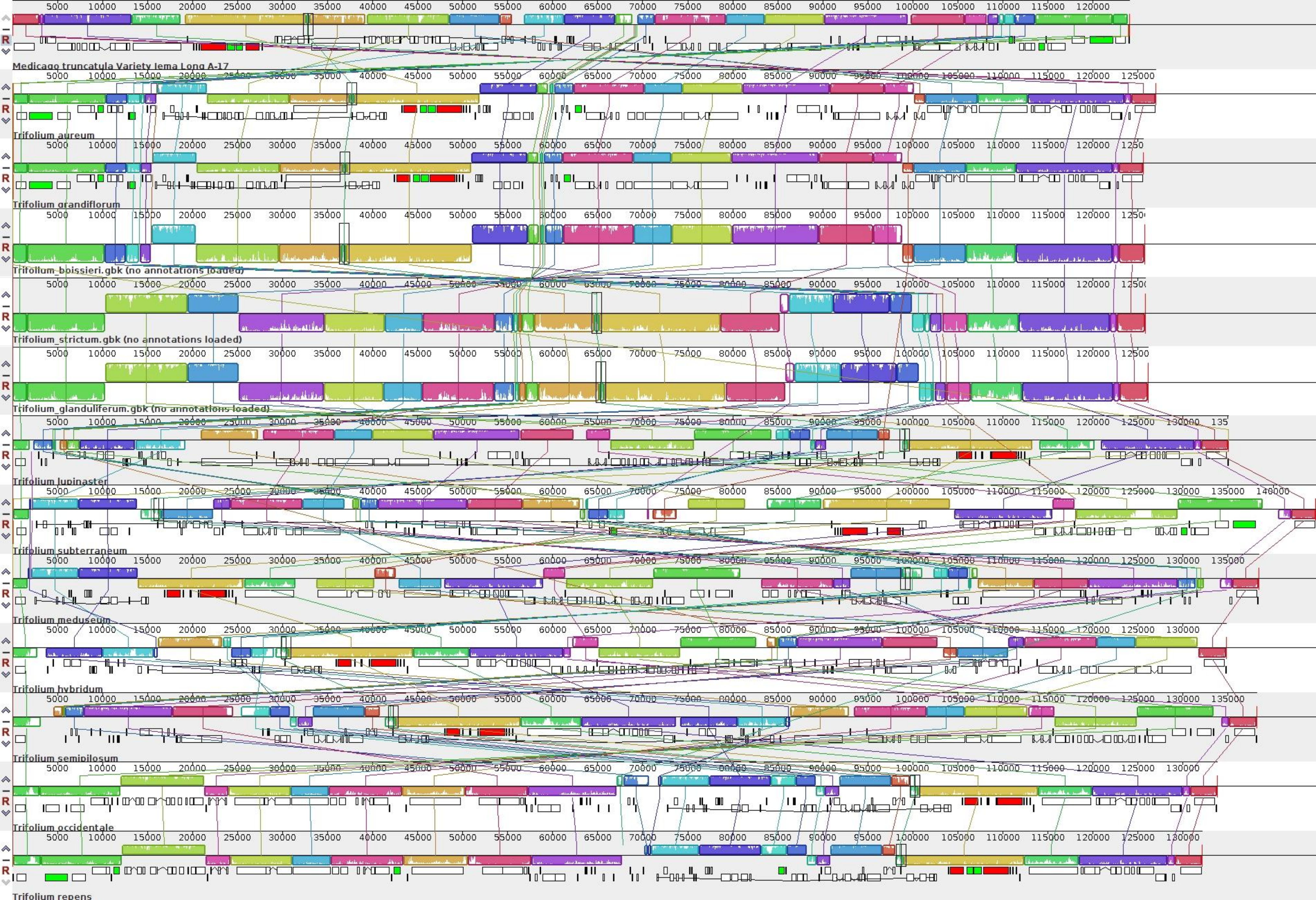

Supplement: Additional file 1: — Genome alignments of plastomes reported in this paper using MAUVE. [file 12862_2014_228_MOESM1_ESM.pdf]
